# Reproductive factors and risk of oesophageal and gastric cancer in the Million Women Study cohort

**DOI:** 10.1038/bjc.2011.525

**Published:** 2011-11-29

**Authors:** J Green, A Roddam, K Pirie, O Kirichek, G Reeves, V Beral

**Affiliations:** 1 Cancer Epidemiology Unit, University of Oxford, Richard Doll Building, Roosevelt Drive, Oxford OX3 7LF, UK; 2 Center for Observational Research, Amgen Ltd, 1 Uxbridge Business Park, Uxbridge UB8 1DH, UK

**Keywords:** oesophageal cancer, gastric cancer, menarche, menopause, parity, cohort

## Abstract

**Background:**

Hormonal factors may influence risk for upper gastrointestinal cancers in women. We examined risk of oesophageal and gastric cancers in relation to reproductive factors in a large UK cohort, the Million Women Study.

**Methods:**

Among 1 319 409 women aged on average 56 years at recruitment, 1186 incident cancers of the oesophagus and 1194 of the stomach were registered during 11.9 million person-years’ observation. Adjusted relative risks (RRs) and 95% confidence intervals (CIs) were estimated using Cox proportional hazards models.

**Results:**

Risks of both oesophageal and gastric cancer were significantly higher in postmenopausal than in pre- or peri-menopausal women (RRs 1.46, 1.07–2.00 and 1.59, 1.15–2.20, respectively; *P*⩽0.01 for both); and, among postmenopausal women, risk was higher the younger women were at menopause (RR, 95% CI per 5 years younger at menopause 1.18, 1.05–1.34 for oesophageal cancer and 1.18, 1.04–1.34 for stomach cancer, *P*_trend_=0.01 for both). For factors relating to childbearing, including women's age at first birth, their number of children, and breastfeeding history, the only significant association was a higher risk of oesophageal cancer in nulliparous, compared with parous, women (RR 1.31, 1.11–1.55; *P*=0.002). When risks for squamous cell and adenocarcinomas of the oesophagus were compared, most did not differ significantly, but statistical power was limited.

**Conclusion:**

Both oesophageal and gastric cancer risks appeared to be related to menopausal status and age at menopause, but there was little consistent evidence for associations with factors related to childbearing.

Cancers of the upper gastrointestinal tract are associated with significant morbidity and mortality. Cancers of the oesophagus and of the stomach are both more common in men than in women and it has been suggested that this may reflect protection offered by exposure to high levels of oestrogens (Lagergren and Nyrén, 1998; Rutegård *et al*, 2010a); hence there is interest in the possible role played by hormonal factors, including those related to menarche and menopause and to childbearing, in determining risk for these cancers (Duell *et al*, 2010; Freedman *et al*, 2010; Rutegård *et al*, 2010b; Bodelon *et al*, 2011). Published evidence suggests that risks for cancers of the oesophagus and stomach are reduced in women taking hormone therapy for the menopause (Green *et al*, 2011). For reproductive factors, the epidemiological evidence is inconsistent, and results from prospective studies are limited by small numbers of cancers.

We report here relationships between reproductive factors and incident cancer of the oesophagus and stomach, by histological type and subsite, in a large UK prospective study of middle-aged women.

## Materials and methods

### Data collection, follow-up and definitions

A total of 1.3 million women aged 50–64 were recruited into the Million Women Study in 1996–2001, completing a recruitment questionnaire about reproductive factors (including age at menarche, menopausal status and, if relevant, age at menopause, number of full-term pregnancies and woman's age at birth of each child, and breastfeeding history), sociodemographic factors and other personal characteristics, including use of hormone therapy for the menopause (HT) and of hormonal contraceptives. Details of the study design and methods are described elsewhere (Reeves *et al*, 2009) and study questionnaires can be viewed at http://www.millionwomenstudy.org. All study participants have a unique National Health Service (NHS) number, and are followed via record linkage (using this number and other personal details) to the NHS Central Registers, so that cancer registrations and deaths are routinely notified to the study investigators. The Central Registers provide information on the date of each such event and code the cancer site and cancer morphology using the 10th revision of the WHO International Classification of Diseases (ICD10). Register follow-up is over 99% complete. All participants gave their written consent to take part in the study and ethical approval was provided by the Oxford and Anglia Multi-Centre Research Ethics Committee.

### Outcome, exposure and adjustment variables

The main endpoints for these analyses are oesophageal cancer (ICD-10 C15) and gastric cancer (ICD-10 C16). Cases of oesophageal cancer were further classed, where possible, as adenocarcinoma (ICD-O morphology codes 8140–8573) or squamous cell carcinoma (ICD-O 8050–8082). Adenocarcinomas of the stomach were classed, where possible, as cancers of the gastric cardia (C16.0) and of specified non-cardia sites (C16.1–16.5). Exposure and adjustment variables for the analyses were derived from information provided at baseline. Women were classed as pre/peri-menopausal if they reported still having periods at the time of recruitment, and as postmenopausal if they reported having had either a natural menopause, or a bilateral oophorectomy. Women who reported having had a hysterectomy were excluded from analyses of menopausal factors; and, in order to study the effect of menopause without additional exogenous hormones, all analyses of menopausal factors were further restricted to never users of HT (and thus women whose menopausal status and age at menopause were masked by use of HT before natural menopause were not included in these analyses). Analyses of age at menopause were restricted to women with known age at natural menopause.

### Statistical analyses

Women diagnosed with any invasive cancer other than non-melanoma skin cancer (ICD10 C44) before recruitment were excluded from the analyses. For the remaining women, person-years were contributed from recruitment until the earliest of date of diagnosis with oesophageal or gastric cancer, date of death, end of follow-up (31 December 2007 for all regions, except for Oxford, Thames, North West (Mersey) and West Midlands (30 June 2008) and East Anglia and Scotland (31 December 2008)), or diagnosis with any cancer other than oesophageal or gastric cancer (or non-melanoma skin cancer) during the follow-up period.

Cox proportional hazards models with attained age as the underlying time variable were used to estimate the relative risks (RRs) of developing oesophageal or gastric cancer in relation to age at menarche (<13, 13–14, 15+ years old), menopausal status (pre/perimenopausal, postmenopausal; with and without censoring at age 55 years), age at natural menopause (50+, 45–49, <45 years old), type of menopause (natural, bilateral oophorectomy), parity (nulliparous, parous) and, within parous women, by number of full-term pregancies (1, 2, 3+), age at first birth (<20, 20–24, 25–29, 30+ years), and breastfeeding of at least one child (never, ever). Women with missing data for an exposure of interest were excluded from the relevant analyses. Within the analyses, adjustment for region of residence (10 cancer registry areas in the UK) and quintiles of socio-economic status (using the Townsend deprivation index (Townsend, Phillimore and Beattie, 1988) was dealt with by using a stratified model, and relevant terms were included in the model for adjustment by body mass index (<22.5, 22.5–24.9, 25.0–29.9, 30.0–34.9, 35 or more kg m^−2^), strenuous physical activity (⩽ once per week, more than once per week), smoking status (never, past, current 1–4, 5–9, 10–14, 15–19, 20+ cigarettes per day), alcohol intake (none, 1–2, 3–6, 7–14, 15+ units per week), use of oral contraceptives (ever, never) and, where appropriate, use of hormone therapy for the menopause (never, past, current). All analyses were mutually adjusted for reproductive factors.

Women with missing values for a given adjustment variable were assigned to a separate category for that variable, and the effect of this strategy was explored by conducting sensitivity analyses among women with known values for all adjustment variables. Statistical tests for heterogeneity between categories and for trend in RR across categories were performed using likelihood ratio tests. A competing risks model (Lunn and McNeil, 1995) was used to assess heterogeneity between results for oesophageal squamous cell and adenocarcinomas and between results for gastric cardia and non-cardia cancers. All analyses used the STATA version 11.1 computing package (StataCorp LP, College Station, TX, USA).

## Results

In all, 1 319 409 women, with a mean age at recruitment of 56.2 (s.d. 4.9) years, were included in the analyses. During a total of 11.9 million woman-years of observation (mean 9.1 years per woman), 1186 incident cancers of the oesophagus and 1194 incident cancers of the stomach were reported, with an average age at diagnosis of about 59 years. Of the oesophageal cancers, 49% (578/1186) were specified as squamous cell carcinoma and 34% (399/1186) as adenocarcinoma, with the remaining 17% (209/1186) not specified histologically. The majority of cancers of the stomach were specified as adenocarcinomas (81% 971/1194), with most of the remaining 233 cancers being of unspecified histology. Of gastric adenocarcinomas, 29% (283/971) were coded to the cardia and 26% (249/971) to specified non-cardia sites, with no site within the stomach specified for 44% (430/971: 9 stomach cancers were of overlapping sites).

Table 1 shows characteristics at recruitment of women included in the analyses, and details of follow-up, by selected categories of reproductive factors. Women with later age at menarche and those with earlier age at menopause were, on average, of lower socioeconomic status, more likely to smoke, and less likely to be obese than women with early menarche or later menopause, respectively. Nulliparous women differed from parous women in being, on average, of higher socioeconomic status, more likely to drink at least 2 units of alcohol per day, less likely to be obese or to smoke, more likely to be physically active and markedly less likely than parous women to have ever used oral contraceptives (45% *vs* 61% for nulliparous and parous, respectively).

Table 2 shows the relationships between reproductive factors and risk of all oesophageal and all gastric cancers. No significant association was seen for either cancer with age at menarche. Compared with pre- or peri-menopausal women, women who were postmenopausal at recruitment had significantly higher risks of both oesophageal and gastric cancer (RRs 1.46, 95% confidence interval (CI) 1.07–2.00 and 1.59, 1.15–2.20, respectively). This analysis was repeated censoring women at age 55 years; after this age the great majority of the women not yet menopausal at recruitment would have reached their menopause. The results obtained were consistent with those in the main analysis, although for much smaller numbers of cases (RRs for postmenopausal *vs* pre- or peri-menopausal women: 2.44, 1.11–5.28 for oesophageal cancer (29 cases) and 1.61, 0.75–3.44 for gastric cancer (27 cases)). For both cancers, risk in postmenopausal women was higher, the younger women were at menopause (RRs per 5 years younger at menopause, 1.18, 95% CI 1.05–1.34 for oesophageal cancer and 1.18, 1.04–1.34, for gastric cancer; *P* for trend=0.01 for each cancer). No difference in risk was seen for either cancer between women with natural menopause and those with bilateral oophorectomy. For childbearing factors, only one significant association was observed. Risk of oesophageal, but not of gastric, cancer was significantly higher in nulliparous than in parous women (RRs 1.31, 1.11–1.55, *P*=0.002, and 1.01, 0.84–1.22, *P*=0.9, respectively). Within parous women, neither cancer was associated with number of full-term pregnancies, age at first birth nor with breastfeeding history.

Table 3 shows the results for oesophageal cancer separately for squamous cell carcinoma and for adenocarcinoma. Significant differences in RR between the two histological types were seen in relation to age at menarche, where a significant decrease in risk with increasing age at menarche was seen for adenocarcinoma (RR per year older, 0.89, 0.82–0.95) but not for squamous cell carcinoma (1.02, 0.97–1.09; *P* for difference in trend, 0.003); and for parity, where the increase in risk in nulliparous compared with parous women was shown to be confined to squamous cell carcinoma (RR 1.60, 1.28–2.00, compared with 0.86, 0.61–1.21 for adenocarcinoma; *P* heterogeneity=0.003). An association with age at menopause was seen for squamous cell carcinoma (RR per 5 years younger at menopause, 1.32, 1.11–1.56) but not for adenocarcinoma (0.98, 0.78–1.23); the difference between the 2 types was of borderline statistical significance (*P* heterogeneity, 0.04).

For gastric cancer, no substantial differences were seen in relation to reproductive factors between specified cardia and non-cardia cancers, although numbers were limited (Table 4).

There were no material differences between the results presented here and those for sensitivity analyses restricted to women with known values for all adjustment variables; nor, for the non-menopause exposures, between results for all women and those from analyses restricted to never users of HT.

## Discussion

In this large prospective analysis, risks of both oesophageal and gastric cancers were higher in postmenopausal than in pre- or peri-menopausal women of a similar age, and, among postmenopausal women, higher for women with an earlier age at menopause. Risk of oesophageal cancer was higher in nulliparous than in parous women, but no other significant associations were seen between risk of either cancer and factors related to childbearing.

Few differences in risk were seen for oesophageal cancers with different histology or for gastric cancers at different subsites. The association between parity and risk of oesophageal cancer was greater for squamous cell than for adenocarcinoma; and age at menarche was significantly associated with adenocarcinoma, but not squamous cell carcinoma, of the oesophagus. No significant differences in risk associated with reproductive factors were seen between adenocarcinomas of the gastric cardia and of non-cardia sites.

Previous epidemiological studies have provided little evidence for strong or consistent relationships between menstrual and reproductive factors and upper gastrointestinal tract cancers. No consistent associations have been found between age at menarche and risk of oesophageal cancer (case–control studies, Gallus *et al*, 2001; Cronin-Fenton *et al*, 2010; cohort studies, Freedman *et al*, 2010; Bodelon *et al*, 2011) or of gastric cancer (case–control studies, Palli *et al*, 1994; Inoue *et al*, 2002; Frise *et al*, 2006; cohort studies, Heuch and Kvåle, 2000; Freedman *et al*, 2007; Persson *et al*, 2008; Duell *et al*, 2010; Freedman *et al*, 2010). There is little published evidence on risk of either cancer by menopausal status, and most studies have had insufficient numbers to compare risk in pre- and postmenopausal women of a similar age.

Some previous studies have found, as we did, significant trends toward lower risk of oesophageal (Gallus *et al*, 2001; Freedman *et al*, 2010) and of gastric (La Vecchia *et al*, 1994; Palli *et al*, 1994; Freedman *et al*, 2007) cancers with increasing age at menopause, although others have not (Heuch and Kvåle 2000; Inoue *et al*, 2002; Persson *et al*, 2008; Cronin-Fenton *et al*, 2010; Bodelon *et al*, 2011). In two cohort studies, an increased risk of gastric cancer has been reported in women with unilateral or bilateral oophorectomy compared with those with natural menopause (Freedman *et al*, 2007; Duell *et al*, 2010) We found no difference in risk of gastric or oesophageal cancer between women with natural menopause and those with bilateral oophorectomy, and similarly no substantial differences were reported in the NIH-AARP cohort (Freedman *et al*, 2010). These comparisons are, however, based on limited numbers of cases with oophorectomy, both in our and in other studies.

Published evidence is insufficient to draw conclusions about risk of oesophageal cancer in relation to menstrual factors by histological type. We found a lower risk associated with older age at menarche for adenocarcinoma but not for squamous cell carcinoma of the oesophagus. A similar pattern of risk was seen in the NIH-AARP cohort (Freedman *et al*, 2010), although in that study the lower risk with older age at menarche for adenocarcinoma was not statistically significant (and the group of oesophageal adenocarcinomas included some adenocarcinomas of the gastric cardia). This association could reflect residual confounding by body mass index, as lower body mass index is associated both with older age at menarche, and with lower risk of adenocarcinoma, but higher risk of squamous cell carcinoma, of the oesophagus (Reeves *et al*, 2009).

For factors related to childbearing, published studies have found no consistent associations with parity for either oesophageal (Gallus *et al*, 2001; Lagergren and Jansson, 2005; Cronin-Fenton *et al*, 2010; Freedman *et al*, 2010; Bodelon *et al*, 2011) or gastric cancer (Palli *et al*, 1994; Heuch and Kvåle, 2000; Inoue *et al*, 2002; Frise *et al*, 2006; Freedman *et al*, 2007, 2010; Bahmanyar *et al*, 2008; Persson *et al*, 2008; Duell *et al*, 2010). Ever breastfeeding was associated with lower risk of adenocarcinomas of the oesophagus and oesophago-gastric junction in the pooled analysis of case–control studies by Cronin-Fenton *et al* (2010), but no association was found in three prospective studies of gastric cancer (Heuch and Kvåle, 2000; Freedman *et al*, 2007; Bahmanyar *et al*, 2008). Our finding of an increased risk for squamous cell carcinoma of the oesophagus in nulliparous compared with parous women may be a chance finding, particularly because we – like others – saw no associations by number of children in parous women for this or any other cancer type examined.

Strengths of the present study include its large size; the prospective determination of exposure and of adjustment factors, minimising risk of recall bias; and full follow-up through reliable disease registries for incident cancers. There is increasing evidence for differences between squamous cell and adenocarcinoma in relation to some risk factors (Reeves *et al*, 2007; Bosman *et al*, 2010), but the few significant differences we observed in cancer risk by histological type in relation to some reproductive factors are difficult to interpret. We had incomplete data on both histological type and (for gastric cancer) subsite, but this should not have substantially biased the results as there is no reason to expect that this information is missing for reasons related to reproductive history. The study relies on self-report of reproductive factors; while some, such as parity, age at first birth and age at menopause are generally well-reported, this is less so for age at menarche (Cairns *et al*, 2011). If errors in recalled age are at random, they are likely to lead to underestimation of the associated cancer risks (Clarke *et al*, 1999).

The strongest and most consistent associations with cancer risk in our study were with menopausal status and age at menopause. Risks of both oesophageal and gastric cancers were higher in postmenopausal than in pre- or peri-menopausal women, and, among postmenopausal women, risks were higher the younger the women were at menopause. The association between menopause and upper gastrointestinal cancer risk may be related to endogenous sex hormone levels, particularly because postmenopausal women taking hormone therapy for the menopause have lower risk of these cancers than women who are not (Green *et al*, 2011). Serum oestradiol levels fall rapidly at the time of the menopause (Rannevik *et al*, 1995) and hormone therapy involves supplementation with exogenous oestrogens. Thus, the changes in risk associated with menopausal status and with hormone therapy use are consistent with an hypothesis that exposure to oestrogens reduces the risk of oesophageal and gastric cancer.

## Figures and Tables

**Table 1:** Characteristics of women included in this analysis, and of follow-up, by selected reproductive factor categories

	**All women**	**Age at menarche**		**Age at menopause^a^**		**Parity**	
**Number of women**	**1 319 409**	**⩽13 Years 806 948**	**14+ Years 485 230**	**<50 Years 168 875**	**50+ Years 236 050**	**Nulliparous 142 622**	**Parous 1 174 497**
*Characteristics at recruitment*							
Age, years (mean (s.d.))	56.2 (4.9)	55.9 (4.8)	56.6 (4.9)	57.6 (5.1)	58.6 (4.4)	56.2 (5.1)	56.1 (4.8)
Socioeconomic status, lowest quintile (%)	19.9	18.5	22.0	24.1	18.4	17.9	20.1
Current smoker (%)	20.5	19.5	22.2	26.8	15.1	17.4	20.9
Alcohol 14+ units per week (%)	6.2	6.3	6.1	5.0	5.0	8.5	5.9
Body mass index >30 kg m^−2^ (%)	18.0	20.2	14.1	18.2	19.1	16.4	18.2
Strenuous exercise >1/week (%)	20.9	21.3	20.3	18.9	21.1	22.1	20.8
Hormone therapy (HT), current use (%)	33.5	33.5	33.4	N/A	N/A	31.7	33.7
Oral contraceptives, ever use (%)	59.3	59.9	58.5	49.9	44.4	45.2	61.1
Nulliparous (%)	10.8	11.2	10.0	13.6	11.6	100	0
Parity (mean (s.d.), in parous women)	2.4 (1.1)	2.4 (1.0)	2.4 (1.1)	2.4 (1.1)	2.4 (1.1)	N/A	2.4 (1.1)
Ever breastfed (%, in parous women)	67.8	68.1	67.5	65.8	70.6	N/A	67.8
							
*Follow-up*							
Person years (millions)	11.9	7.3	4.4	1.5	2.1	1.3	10.6
Incident cancers (*n*)							
Oesophagus	1186	720	433	258	247	163	1021
Oesophagus, squamous cell	578	324	238	144	101	97	480
Oesophagus, adenocarcinoma	399	272	117	74	96	36	362
Stomach	1194	701	465	236	239	125	1067
Adenocarcinoma, gastric cardia	283	176	97	60	48	29	253
Adenocarcinoma, non-cardia	249	150	92	47	55	28	219

Abbreviation: N/A=not applicable.

a Restricted to women with known age at menopause who have never used HT (see Materials and Methods).

**Table 2:** RRs and 95% CIs for incident cancers of the oesophagus and stomach in relation to reproductive factors

	**Oesophagus**		**Stomach**	
	***n* Cases**	**RR, 95% CI**	***n* Cases**	**RR, 95% CI**
*Menarche and menopause*				
Age at menarche				
<13 Years	444	1.11, 0.97–1.26	418	1.00, 0.88–1.14
13–14 Years	501	Ref	505	Ref
15+ Years	208	0.96, 0.82–1.13	243	1.10, 0.95–1.29
Trend per year older		0.96, 0.93–1.00		1.02, 0.98–1.06
	*P*_trend_=0.1		*P*_trend_=0.3	
				
Menopausal status				
Pre/perimenopausal	59	Ref	53	Ref
Postmenopausal	539	1.46, 1.07–2.00	526	1.59, 1.15–2.20
	*P*_heterogeneity_=0.01		*P*_heterogeneity_=0.004	
				
Age at menopause				
50+ Years	247	Ref	239	Ref
45–49 Years	180	1.32, 1.09–1.61	160	1.26, 1.03–1.54
<45 Years	78	1.34, 1.03–1.74	76	1.35, 1.04–1.76
Trend per 5 years younger		1.18, 1.05–1.34		1.18, 1.04–1.34
	*P*_trend_=0.01		*P*_trend_=0.01	
				
Type of menopause				
Natural	521	Ref	503	Ref
Bilateral oophorectomy	18	0.97, 0.60–1.57	23	1.22, 0.79–1.88
	*P*_heterogeneity_=0.9		*P*_heterogeneity_=0.4	
				
*Childbearing*				
Parity				
Nulliparous	163	1.31, 1.11–1.55	125	1.01, 0.84–1.22
Parous	1021	Ref	1067	Ref
	*P*_heterogeneity_=0.002		*P*_heterogeneity_=0.9	
				
Number of full-term pregnancies^a^				
1	164	0.94, 0.77–1.14	201	1.22, 1.02–1.47
2	445	Ref	425	Ref
3+	412	1.00, 0.87–1.15	441	1.03, 0.90–1.19
Trend per birth		1.02, 0.94–1.09		0.97, 0.90–1.04
	*P*_trend_=0.7		*P*_trend_=0.3	
				
Age at first birth^a^				
<20 Years	124	0.81, 0.66–0.99	156	1.01, 0.84–1.21
20–24 Years	511	Ref	515	Ref
25–29 Years	268	0.97, 0.83–1.13	261	0.98, 0.84–1.14
30+ Years	87	0.92, 0.73–1.17	97	1.09, 0.87–1.36
Trend per 5 y older		1.02, 0.94–1.12		1.02, 0.93–1.14
	*P*_trend_=0.6		*P*_trend_=0.7	
				
Breastfeeding^a^				
Never	260	Ref	243	Ref
Ever	521	0.89, 0.76–1.04	529	1.03, 0.88–1.12
	*P*_heterogeneity_=0.2		*P*_heterogeneity_=0.7	

Abbreviations: CI=confidence intervals; ref=reference; RRs=relative risks.

RRs stratified by age, region and socioeconomic status and adjusted for body mass index, smoking, alcohol, strenuous exercise, use of oral contraceptives and, as appropriate, for all other reproductive factors and for use of hormone therapy for the menopause (HT).

Analyses of all menopausal factors restricted to never HT users; analyses of age at menopause restricted to women with natural menopause.

a Restricted to parous women.

**Table 3:** RRs and 95% CIs for squamous cell and adenocarcinomas of the oesophagus in relation to reproductive factors

	**Oesophagus, squamous cell carcinoma**		**Oesophagus, adenocarcinoma**		
	***n* Cases**	**RR, 95% CI**	***n* Cases**	**RR, 95% CI**	**Test for difference, squamous cell and adenocarcinoma (*P*)**
*Menarche and menopause*					
Age at menarche					
<13 Years	184	0.95, 0.78–1.15	178	1.29, 1.04–1.60	0.003
13–14 Years	259	Ref	158	Ref	
15+ Years	119	1.04, 0.84–1.30	53	0.80, 0.59–1.10	
Trend per year older		1.02, 0.97–1.09		0.89, 0.82–0.95	
	*P*_trend_=0.4		*P*_trend_=0.001		
					
Menopausal status					
Pre/perimenopausal					
Postmenopausal	31	Ref	20	Ref	0.4
	258	1.65, 1.08–2.53	183	1.18, 1.69–2.04	
	*P*_heterogeneity_=0.02		*P*_heterogeneity_=0.5		
					
Age at menopause					
50+ Years	101	Ref	96	Ref	0.04
45–49 Years	102	1.69, 1.28–2.24	55	1.12, 0.80–1.57	
<45 Years	42	1.65, 1.14–2.39	19	0.89, 0.54–1.47	
Trend per 5 years younger		1.32, 1.11–1.56		0.98, 0.78–1.23	
	*P*_trend_=0.002		*P*_trend_=0.9		
					
Type of menopause					
Natural	253	Ref	176	Ref	0.4
Bilateral oophorectomy	5	0.59, 0.24–1.14	7	1.13, 0.52–2.46	
	*P*_heterogeneity_=0.2		*P*_heterogeneity_=0.8		
			
*Childbearing*					
Parity					
Nulliparous	97	1.60, 1.28–2.00	36	0.86, 0.61–1.21	0.003
Parous	480	Ref	362	Ref	
	*P*_heterogeneity_<0.0001		*P*_heterogeneity_=0.4		
					
Number of full-term pregnancies^a^					
1	78	0.91, 0.69–1.22	55	0.86, 0.61–1.19	0.3
2	206	Ref	168	Ref	
3+	196	1.11, 0.91–1.37	139	0.84, 0.66–1.07	
Trend per birth		1.08, 0.97–1.20		0.95, 0.84–1.08	
	*P*_trend_=0.2		*P*_trend_=0.5		
					
Age at first birth^a^					
<20 Years	59	0.87, 0.65–1.16	40	0.69, 0.48–0.97	0.6
20–24 Years	224	Ref	194	Ref	
25–29 Years	135	1.12, 0.90–1.40	88	0.84, 0.65–1.09	
30+ Years	45	1.11, 0.80–1.54	31	0.85, 0.57–1.25	
Trend per 5 years older		1.10, 0.96–1.35		1.00, 0.86–1.17	
	*P*_trend_=0.2		*P*_trend_=0.9		
					
Breastfeeding^a^					
Never	120	Ref	105	Ref	0.1
Ever	256	0.97, 0.77–1.22	168	0.75, 0.58–0.97	
	*P*_heterogeneity_=0.8		*P*_heterogeneity_=0.03		

Abbreviations: CI=confidence intervals; ref=reference; RRs=relative risks.

RRs stratified by age, region and socioeconomic status and adjusted for body mass index, smoking, alcohol, strenuous exercise, use of oral contraceptives and, as appropriate, for all other reproductive factors and for use of hormone therapy for the menopause (HT). Analyses of all menopausal factors restricted to never HT users; analyses of age at menopause restricted to women with natural menopause.

a Restricted to parous women.

**Table 4:** RRs and 95% CIs for adenocarcinomas of the stomach, by site, in relation to reproductive factors

	**Adenocarcinomas of gastric cardia**		**Adenocarcinomas of other gastric sites**		
	***n* Cases**	**RR, 95% CI**	***n* Cases**	**RR, 95% CI**	**Test for difference, Cardia v non-cardia (*P*)**
*Menarche and menopause*					
Age at menarche					
<13 Years	115	1.23, 0.95–1.60	83	0.94, 0.71–1.25	0.4
13–14 Years	108	Ref	111	Ref	
15+ Years	49	1.05, 0.75–1.47	47	0.95, 0.68–1.34	
Trend per year older		0.95, 0.87–1.03		1.01, 0.92–1.10	
	*P*_trend_=0.2		*P*_trend_=0.9		
					
Menopausal status					
Pre/perimenopausal					
Postmenopausal	14	Ref	10	Ref	0.5
	116	1.20, 0.63–2.31	117	1.64, 0.79–3.41	
	*P*_heterogeneity_=0.6		*P*_heterogeneity_=0.2		
					
Age at menopause					
50+ Years	48	Ref	55	Ref	0.3
45–49 Years	38	1.44, 0.94–2.22	32	1.10, 0.71–1.71	
<45 Years	22	1.80, 1.07–3.01	15	1.15, 0.64–2.06	
Trend per 5 years younger		1.35, 1.05–1.74		1.08, 0.82–1.43	
	*P*_trend_=0.02		*P*_trend_=0.6		
					
Type of menopause					
Natural	112	Ref	109	Ref	1.0
Bilateral oophorectomy	4	0.86, 0.31–2.34	4	1.03, 0.37–2.88	
	*P*_heterogeneity_=0.8		*P*_heterogeneity_=0.9		
					
			
*Childbearing*					
Parity					
Nulliparous	29	1.08, 0.73–1.60	28	1.06, 0.71–1.59	0.9
Parous	253	Ref	219	Ref	
	*P*_heterogeneity_=0.7		*P*_heterogeneity_=0.8		
					
Number of full-term pregnancies^a^					
1	57	1.91, 1.35–2.72	43	1.37, 0.91–2.05	0.2
2	91	Ref	79	Ref	
3+	105	1.08, 0.8–1.44	97	1.23, 0.90–1.68	
Trend per birth		0.86, 0.74–1.00		1.02, 0.87–1.20	
	*P*_trend_=0.04		*P*_trend_=0.8		
					
Age at first birth^a^					
<20 Years	38	0.89, 0.62–1.29	32	1.02, 0.68–1.52	1.0
20–24 Years	133	Ref	108	Ref	
25–29 Years	56	0.86, 0.62–1.18	49	0.88, 0.62–1.25	
30+ Years	21	0.98, 0.61–1.58	22	1.24, 0.77–1.99	
Trend per 5 years older		0.93, 0.83–1.19		1.03, 0.85–1.26	
	*P*_trend_=0.9		*P*_trend_=0.7		
					
Breastfeeding^a^					
Never	64	Ref	48	Ref	0.7
Ever	118	0.91, 0.67–1.26	107	1.07, 0.75–1.52	
	*P*_heterogeneity_=0.6		*P*_heterogeneity_=0.7		

Abbreviations: CI=confidence intervals; HT=hormone therapy; ref=reference; RRs=relative risks.

RRs stratified by age, region and socioeconomic status and adjusted for body mass index, smoking, alcohol, strenuous exercise, use of oral contraceptives and, as appropriate, for all other reproductive factors and for use of HT. Analyses of all menopausal factors restricted to never HT users; analyses of age at menopause restricted to women with natural menopause.

a Restricted to parous women.

## APPENDIX

### Million Women Study Collaborators

*Steering Committee* Emily Banks, Valerie Beral, Ruth English, Jane Green, Julietta Patnick, Richard Peto, Gillian Reeves, Martin Vessey, and Matthew Wallis.

*NHS Breast Screening Centres collaborating in the Million Women Study (in alphabetical order)*Avon, Aylesbury, Barnsley, Basingstoke, Bedfordshire and Hertfordshire, Cambridge and Huntingdon, Chelmsford and Colchester, Chester, Cornwall, Crewe, Cumbria, Doncaster, Dorset, East Berkshire, East Cheshire, East Devon, East of Scotland, East Suffolk, East Sussex, Gateshead, Gloucestershire, Great Yarmouth, Hereford and Worcester, Kent (Canterbury, Rochester, Maidstone), Kings Lynn, Leicestershire, Liverpool, Manchester, Milton Keynes, Newcastle, North Birmingham, North East Scotland, North Lancashire, North Middlesex, North Nottingham, North of Scotland, North Tees, North Yorkshire, Nottingham, Oxford, Portsmouth, Rotherham, Sheffield, Shropshire, Somerset, South Birmingham, South East Scotland, South East Staffordshire, South Derbyshire, South Essex, South Lancashire, South West Scotland, Surrey, Warrington Halton St Helens and Knowsley, Warwickshire Solihull and Coventry, West Berkshire, West Devon, West London, West Suffolk, West Sussex, Wiltshire, Winchester, and Wirral and Wycombe.

*Million Women Study Co-ordinating Centre Staff* Simon Abbott, Miranda Armstrong, Angela Balkwill, Emily Banks, Vicky Benson, Valerie Beral, Judith Black, Kirsty Bobrow, Anna Brown, Diana Bull, Benjamin Cairns, Karen Canfell, Dexter Canoy, James Chivenga, Barbara Crossley, Dave Ewart, Sarah Ewart, Lee Fletcher, Toral Gathani, Laura Gerrard, Adrian Goodill, Jane Green, Lynden Guiver, Michal Hozak, Isobel Lingard, Elizabeth Hilton, Sau Wan Kan, Carol Keene, Oksana Kirichek, Mary Kroll, Nicky Langston, Bette Liu, Maria-Jose Luque, Lynn Pank, Kirstin Pirie, Gillian Reeves, Emma Sherman, Evie Sherry-Starmer, Moya Simmonds, Helena Strange, Siân Sweetland, Alison Timadjer, Sarah Tipper, Joanna Watson, Lucy Wright, and Heather Young.
